# Sure Steps: Key Strategies for Protecting Basketball Players from Injuries—A Systematic Review

**DOI:** 10.3390/jcm13164912

**Published:** 2024-08-20

**Authors:** Yoel Antoranz, Eduardo Sáez de Villarreal, Juan del Campo Vecino, Sergio L. Jiménez-Saiz

**Affiliations:** 1 Department of Physical Education, Sport and Human Movement, Universidad Autónoma de Madrid, 28049 Madrid, Spain; yoel.antoranz@estudiante.uam.es (Y.A.); juan.delcampo@uam.es (J.d.C.V.); 2 Physical Performance Sports Research Center (PPSRC), Universidad Pablo Olavide Sevilla, 41013 Sevilla, Spain; esaesae@upo.es; 3 Sport Sciences Research Centre, Faculty of Education & Sport Sciences and Interdisciplinary Studies, Universidad Rey Juan Carlos, 28942 Fuenlabrada, Madrid, Spain

**Keywords:** injury prevention, basketball, general injuries, ankle sprain, anterior cruciate ligament injury

## Abstract

**Background:** Basketball is a high-intensity sport, which includes actions such as jumping, changes of direction, accelerations, and decelerations, which generates fatigue situations that may increase the risk of injury. Specifically, the joints at greatest risk are the ankle and knee, with ankle sprains and anterior cruciate ligament (ACL) tears being the most prevalent injuries. There are several strategies aimed at reducing the incidence, based on training methods or other prophylactic measures. Therefore, the purpose of the study is to perform a systematic review of the different injury prevention strategies in competitive-level basketball players with respect to general injuries, ankle sprains, and ACL injuries. **Methods:** For this purpose, the PRISMA methodology was applied, performing a search in three databases (PubMed, SPORTDiscus, and Cochrane) between 25 September 2023 and 8 October 2023. **Results:** A total of 964 articles were identified, out of which 283 were duplicates and 644 were discarded. Out of the remaining 37, 23 were excluded because they did not meet the inclusion criteria; therefore, 14 articles were finally included. With respect to general injuries, 8 out of 14 studies reviewed them. Concerning ankle sprains, 7 studies specifically analyzed them. Finally, 3 studies focused on ACL injuries. **Conclusions:** Training programs that combine different contents, known as neuromuscular training, including strength work, stabilization or core, mobility, and agility are the most effective for both general injuries and ACL injuries. For ankle sprains, the most effective measures are training programs based on analytical ankle stability exercises and the use of ankle braces. Adherence to prevention programs is essential, so they can be included as part of the warm-up. Other strategies such as training load control, functional assessment, or rule modification are not used in the included articles, so their effectiveness as prophylactic methods could not be justified.

## 1. Introduction

Basketball is a widely popular sport globally but carries a significant injury risk. Statistics show that one in three young athletes and adults experience injuries requiring medical attention or causing missed practice annually [1,2]. Therefore, creating effective injury prevention programs is crucial [3].

In high-demand sports like basketball, the injury risk is elevated due to intense actions such as jumps, changes of direction (CODs), accelerations, and decelerations [1,4]. Despite the decreasing physical demands from the first to the last quarter of a game, fatigue diminishes joint stability and negatively impacts single-leg postural control, ankle joint position sense, and peroneus longus reaction time, increasing the risk of injury during CODs [5,6,7].

In basketball, the injury rate (injuries/hours of practice) is higher in competition in senior (IRR = 2.02; 95% CI = 1.90, 2.14) and young athletes (IRR = 2.38; 95% CI = 2.22, 2.56). The most common injuries are in the lower limbs, particularly ankle sprains and anterior cruciate ligament (ACL) tears [4,8,9,10,11]. Women are up to three times more likely to suffer ACL injuries due to anatomical, neuromuscular, and hormonal factors [11,12]. The most common injury mechanisms are training overload, landings, or contact with another athlete [4,9,10]. Ankle injury rates are between 1 and 5.2 per 1000 practice hours for both sexes, with 90% being sprains [2]. The most common are those of the external lateral ligament. The main risk factor is a previous injury. Frequent mechanisms include ankle inversion [13]. Recovery from an ACL injury typically spans over 9 months, sometimes extending to 12 months [14,15]. These injuries usually occur without contact, with common mechanisms being internal rotation and valgus of the knee, in extension, during CODs or landings [16,17]. Numerous anatomical and neuromuscular factors are associated with these injuries [18].

Sports injuries are a multifactorial phenomenon [19], so the approach should encompass different prophylactic strategies. The most studied measure in the scientific literature is training programs, although others such as load control, functional assessment, the modification of regulations, or the use of certain equipment are proposed.

Several training methods, such as strength, proprioceptive, stability, and motor control training, have been studied as preventive measures. Neuromuscular training, a term in the scientific literature, groups these methods with agility and movement quality training. Studies show these programs effectively reduce injury incidence [3]. One major problem is adherence, but incorporating various preventive stimuli into warm-ups can generate high adherence and establish a useful routine for athletes [3].

Another preventive measure is training load management [3]. Research links match loads to injury rates among NBA players [20], but Ferioli et al. found no association between internal loads and non-contact injuries, suggesting limited predictive reliability [21]. Gabbett and others argue that injuries are more likely when athletes face unfamiliar loads, especially if acute loads significantly exceed chronic (cumulative) loads [22,23].

Another method proposed is functional assessment to identify deficits, asymmetries, and imbalances, aiming to mitigate intrinsic risk factors and reduce injury risk. The Functional Movement Screen (FMS) is widely utilized for this purpose. While some studies support its validity in assessing injury likelihood [24], others suggest its predictive and preventive abilities are limited [24,25,26,27,28]. The reliability of this system remains unclear.

Apart from training control, measures also include equipment modifications. Specialized sports equipment and protective gear, like footwear and ankle tapes commonly used in basketball, are employed to reduce injury rates [3].

Additionally, there are ongoing efforts to develop advanced technology-based models. These include mathematical algorithms based on motion patterns [29] and even robotic systems [30]. Advanced motion capture systems [31,32] are also being explored, although further development and research are needed in these areas.

After reviewing the literature, it has been found that there are different strategies to reduce injury rates. Therefore, the aim of this work is to carry out a systematic review to establish the current evidence regarding injury prevention programs, based on training methods or other strategies, and their effectiveness in reducing (1) general injuries, (2) ankle sprains, and (3) ACL injuries in non-recreational basketball players, those at the competitive level.

## 2. Materials and Methods

### 2.1. Study Design and Search Strategy

A systematic review was conducted following the guidelines of the Preferred Reporting Items for Systematic Reviews and Meta-Analyses (PRISMA) Statement [33]. The search was conducted between 25 September 2023 and 8 October 2023. The PubMed, SPORTDiscus, and Cochrane Central Register of Controlled Trials (Cochrane) databases were reviewed. The search engine used was the following: (“anterior cruciate ligament” OR “ACL” OR “ankle sprain” OR “injury*”) AND (“prevent*” OR “training load”) AND “basketball”. The filters “Abstract/Title” in SPORTDiscus and Pubmed and “Abstract/Title/Keywords” in Cochrane and the criterion that all the papers were in the English language were used to narrow the search. In addition, the filter “scientific publications” was selected in SPORTDiscus.

### 2.2. Inclusion Criteria

The studies included in the review were selected based on the following criteria: (A) Published scientific articles. (B) Randomized controlled trials or prospective studies in which there is a comparison with other groups. (C) The study population was both male and female basketball players, for school and senior age, if it was competitive or if the study population included other sports, and if the results were shown separately for each sport. (D) Intervention measures aimed to reduce injury incidence. (E) The training program, including various parameters such as frequency, duration, and exercise types, as well as the measures implemented (equipment, regulations, and training load control), were comprehensively detailed and explained. (F) Data on injury incidence were accessible, along with information on hazard ratios (HRs), relative risk (RR), odds ratios (ORs), or similar measures. (G) The articles were in English, and both the abstract and full text were accessible.

Prospective studies were included; given that, in high-level teams, it is complicated to obtain samples in which a control group can be used or to randomize training programs, prospective or non-randomized studies could also be of interest. Case studies and studies with samples of Paralympic athletes or wheelchair basketball were not included.

If there were doubts about whether to include an article in the review, two parallel actions were taken: Consult with a co-reviewer to ensure impartiality and leverage their expertise. Conduct an independent double review with two independent expert reviewers on the condition that both reviewers agree on its inclusion or exclusion.

### 2.3. Quality Assessment

The studies that were finally included in the review were analyzed with a modified version of the Reach Efficacy Adoption Implementation Maintenance (RE-AIM) framework tool. The original version consists of 31 items; however, for this paper, an adapted version was employed with 10 items specifically designed for the prevention of sports injuries, similar to the one used by Taylor et al. [34,35] (see Appendix A, Table A1).

### 2.4. Data, Variables, and Measures Reviewed

All data used for the review were extracted directly from the studies reviewed without further analysis. The main variables sought in the studies were both injuries, mainly ankle sprains and ACL injuries, and their severity. The usual measures were the injury index (number of injuries/1000 h of exposure) and others to compare injury indices between groups such as the hazard ratio (HR), relative risk (RR), odds ratio (OR), Absolute Risk Ratio (ARR), and Number Needed to Treat (NNT).

## 3. Results

**Figure 1:**
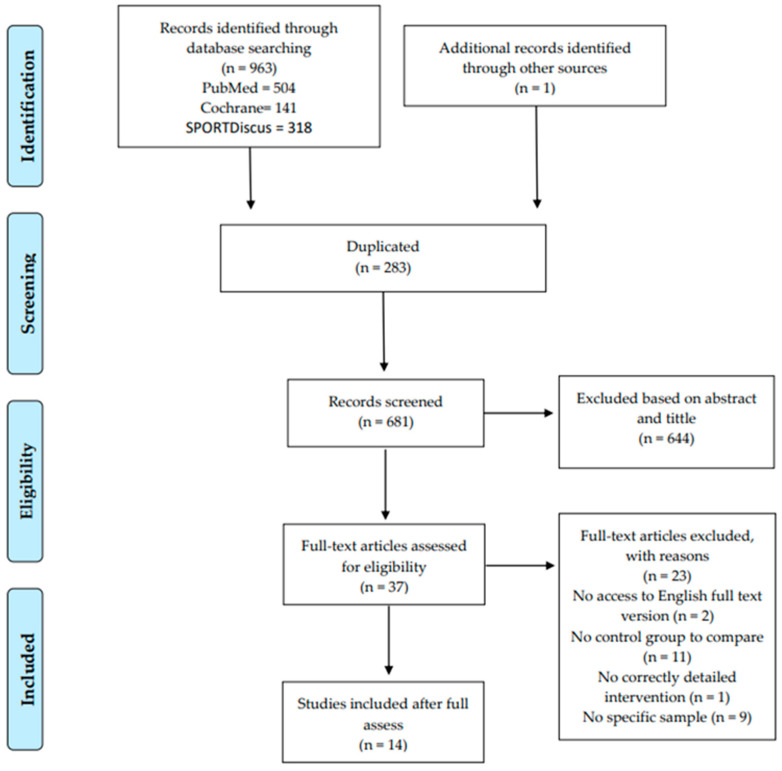
Flow diagram of study identification.

A total of 964 results in the literature were identified in the search, distributed across different databases: 504 in PubMed, 141 in Cochrane, and 318 in SPORTDiscus, with 1 additional article found in the systematic review by Taylor et al. [35]. After removing 283 duplicates, the remaining 681 records were further assessed. Out of these, 644 were excluded. Subsequently, we conducted a thorough evaluation of the remaining 37, leading to the exclusion of 23 due to non-compliance with one or more inclusion criteria (see Figure 1).

The remaining 14 articles met the inclusion criteria [36,37,38,39,40,41,42,43,44,45,46,47,48]. Table 1 shows the included studies, details of the sample, and the intervention performed, as well as the results and conclusions obtained. In some studies, additional variables unrelated to injury incidence or severity, such as performance indicators in various tests or functional assessment results, were analyzed but not considered. Data referring to other sports studied in the studies analyzed were not recorded either. Out of these 14 studies, 11 are specific to basketball and another 3 of them combine players from various sports in the sample. Of the latter 3, only the data referring to basketball players were analyzed. With regard to the search objectives, 8 out of the 14 studies show data on general injuries, 7 of them on ankle sprains, and 3 on ACL. The average quality score of the articles obtained in the RE-AIM was 4.57 out of 10 (see Appendix A).

### 3.1. General Injuries

Out of the eight papers included, six of these show positive results, with five of them using neuromuscular training programs.

Good results were seen with a program based on neuromuscular core, stabilization, and strength training performed 3 days a week in the preseason and 2 days during the season [37] (4.99/1000 h vs. 7.72/1000 h; ARR = 40 [95% CI = 0.92, 4.54]). The work of Bonato et al. showed that even using warm-ups throughout the season that include neuromuscular exercise, in this case, mobility, strength, plyometrics, and agility, can be helpful in reducing injury rates (1.66/1000 h vs. 4.69/1000 h, *p* = 0.012) [39]. The work of Emery et al., in which a program based on strength, agility, stability, and endurance exercises was performed, was also effective in reducing overall injuries to both ankle and knee, even though the program is performed unsupervised, 3 days a week for at least 10 min [48].

The work of Longo et al. also showed that a neuromuscular warm-up such as FIFA 11+, which combines mobility, running, agility, strength, core, and stabilization exercises, can significantly reduce overall injuries (0.95/1000 h vs. 2.16/1000 h; *p* = 0.0004), in lower extremities, acute and severe injuries, and the trunk, hip, and groin. However, there were no significant reductions in knee, ankle, and overuse injuries compared to the control group [43]. Finally, the neuromuscular warm-up program used by Stojanović et al., which included dynamic stretching, strength, stability, balance, and plyometric exercises, effectively succeeded in terms of reducing overall knee injuries (IRR = 0.32, 95% CI = 0.03, 1.78, *p* = 0.07) and overall non-contact lower body injuries (IRR = 0.26, 95% CI = 0.05, 0.98, *p* < 0.001) [47].

Another program based on the jumping and landing technique, performed 2 days a week for 10 min, can significantly reduce overall injury incidence (3.6/1000 h vs. 5.4/1000 h; HR = 0.40 [95% CI = 0.16–0.99]) [36]. Similarly, Hewett et al. [42] showed that a plyometric training program conducted during the preseason for 4–6 weeks significantly reduced ACL injuries in females compared to those who did not participate. This program also included strength training and agility exercises.

Other studies have not shown good results in reducing overall injuries. The work by Emery et al. focusing on stability and mobility work also found no significant reductions in overall (RR = 0.8 [95% CI 0.57–1.11]) or lower limb (RR = 0.83 [95% CI 0.57–1.19]) injury rates [41].

### 3.2. Ankle Sprains

There are seven studies included in the review that analyze ankle sprains, five of them using training methods and two using sports equipment.

With regard to training methods, the Cumps et al. study showed significant risk reductions in the overall group (RR = 0.30 [95% CI: 0.11–0.84]) [40] after performing a 22-week ankle joint stability training program using stabilization semi-globes. Analytical ankle stabilization exercises (such as balancing and landing) without equipment on a multi-station circuit performed throughout the season are also effective in reducing injury incidence relative to a control group (1.66/1000 h vs. 4.69/1000 h, *p* = 0.012; OR = 0.355, [95% CI = 0.151–0.835, *p* = 0.018]) [49]. However, another training program focusing on analytical ankle stability using a stabilization platform (Wobble Board) did not yield significantly positive results [41].

Regarding neuromuscular training, the work of Bonato et al. [39], which involved significant reductions in terms of overall injuries, did not obtain the same results in terms of ankle sprains (*p* = 0.507). In contrast, the work of Stojanović et al. obtained results opposite to the previously mentioned work. In this case, a warm-up based on neuromuscular work that included strength, agility, balance, plyometrics, and mobility exercises did lead to a reduction in ankle injury incidence [47].

Other types of injury prevention methods have been analyzed. The use of lace-up ankle braces for a whole season has been shown to be effective with respect to a control group (0.47/1000 h vs. 1.41/1000 h; HR = 0.32 [95% CI = 0.20–0.52; *p* < 0.01]) and even with players with previous sprains (0.83/1000 h vs. 1.79/1000 h; HR = 0.39 [95% CI = 0.17–0.90; *p* < 0.01]) [44]. Shoe type appears to not influence the incidence of ankle sprains [38].

### 3.3. Anterior Cruciate Ligament

Three studies specifically analyze anterior cruciate ligaments.

One of them is the work of Bonato et al. [39], which included strength, mobility, plyometric, and agility exercises. The training program was effective in reducing knee sprains and specifically ACL injuries (*p* = 0.038).

Similarly, Omi et al. [45] based their program on the combination of different training contents such as strengthening exercises, and others such as plyometrics and landings, and stability exercises. Similarly, it has shown very positive results with regard to ACL injuries. In this study, the same sample underwent two periods: an observation and an intervention period. During the intervention period, there was a lower injury incidence, and statistical indicators demonstrated a reduced risk for all ACL injuries (0.1/1000 h vs. 0.25/1000 h; RR = 0.38; [95% CI, 0.17–0.87; *p* = 0.017]; ARR = 0. 032 [95% CI, 0.027–0.037]; and NNT = 31.6 [95% CI, 27.1–37.7]) as for non-contact ACL injuries (0.08/1000 h vs. 0.21/1000 h; RR = 0.37 [95% CI, 0.15–0.92; *p* = 0.026]; ARR = 0.024 [95% CI, 0.020–0.029]; and NNT = 41.3 [95% CI, 34.6–51.3]).

In contrast, the work of Pfeiffer et al. [46] based on a training program of plyometrics, landings, and decelerative movement mechanics yielded negative results. The study involved female basketball, soccer, and volleyball players. The differences between the control and experimental groups in the basketball subgroup were not significant, indicating that this twice-weekly program was ineffective in preventing non-contact ACL injuries.

### 3.4. Sex

The general injury studies analyzed include samples of both sexes, in some cases together and in others only female players, showing positive results in both cases. The work by Emery et al. [41] showed that sex did not modify the effect of the training program, but that, nevertheless, regardless of the study group (control or experimental), the relative risk was higher for women compared to men for general injuries (RR = 1.64 [95% CI; 1.14–2.33]). Another work by Emery et al. also showed that sex had no influence on the effect of a neuromuscular training program on reducing the incidence of ankle sprains [48]. Finally, Hewett et al. [42] found differences in knee injury incidence among trained women, untrained women, and the control group of trained men in a basketball sample.

Regarding ankle sprains, Cumps et al. found no significant differences between men and women when analyzed separately [40]. Eils et al.’s study included both sexes but presented combined results without specifying differences [49]. Similarly, McGuine et al. analyzed the use of ankle braces without separating results by sex, preventing inference on efficacy differences [44].

All the programs analyzed in the present review about ACL injury are conducted with an exclusive sample of women.

### 3.5. Age

Emery et al. (14.0 ± 1.7 years) and Barber Foss et al. (11–18 years) studied general injuries in young players, suggesting multicomponent neuromuscular training is effective for this group. Longo et al. included players of all categories, from under 12 to senior. Stojanovic et al. and Bonato et al. focused on senior female athletes [37,39,43,47,48]. Aerts et al. used a plyometrics, jumping, and landing technique program with male and female players aged 15–41 years [36].

Regarding ankle sprains, Cumps et al. studied athletes in school and university (control group = 18.0 ± 2.7 years; intervention group = 17.7 ± 3.9 years), while Eils et al. focused on senior players from the seventh to the first German division. McGuine et al. used ankle braces on high school athletes (16.0 ± 1.1 years), so the results are specific to that group [40,44,49].

Finally, reviewing the studies analyzing ACL injuries with positive results, the player samples used in both cases are from senior athletes over age [39,45].

### 3.6. Training Methods and Other Injury Prevention Strategies

Neuromuscular training programs are effective in the prevention of all types of injuries and ACL injuries, and specific balance and stability programs are effective in the prevention of ankle sprains. On the other hand, it has been commented that lace-up ankle braces have also been shown to be effective in relation to ankle sprains. Other types of measures or strategies have not been analyzed.

### 3.7. Quality Assessment of the Studies

The evaluation, using the modified 10-item version of RE-AIM, showed a mean of 4.57 out of 10, with values ranging from 2 to 8 points. The values obtained in each study can be consulted in Appendix A, which contains the tables with the individual scores for each of the items.

## 4. Discussion

### 4.1. General Injuries

As could be seen in the analyzed works, multicomponent training based on various contents, known in the literature as neuromuscular training [3], is the most effective for the reduction of general injuries. Strength training is included in all the programs analyzed, although other contents are common in the vast majority such as stability or core work, mobility, or, to a lesser extent, agility and plyometrics. On the other hand, it has been observed that other programs, like those by Aerts et al. and Hewett et al., based on plyometric training, are also effective. The Aerts et al. program included technical jump and landing exercises, along with plyometrics. The Hewett et al. program not only included the mentioned exercises but also involved strength and agility training, making it more similar to the multifaceted programs discussed earlier. Therefore, multicomponent programs are not only effective, but they are also the ones that show the most abundant evidence.

Some of these studies use the prevention program as a warm-up. As stated in the Introduction [3], its inclusion as a warm-up is an appropriate strategy to promote adherence. One of the fundamental aspects should be to try to carry out this type of program throughout the season. The study by Emery et al. [41] did not show good results either. However, the problem in this work was that compliance with the program was low (60.3%). Therefore, in this case, it can also be seen that one of the fundamental variables is adherence to the program, so if it is not maintained over time or is not carried out consistently, it will not be effective in terms of prevention. Low adherence has been postulated as one of the main risk factors that prevent a neuromuscular injury prevention program from being successful [50,51].

Ultimately, multicomponent programs are effective in preventing general basketball injuries and reducing injury rates. These programs should be performed 2–3 days a week, lasting between 10 and 30 min, and should include mainly strength, stability, and mobility work, as well as agility and plyometrics. They can be included as part of the warm-up and be performed in every training session, which will promote adherence to the program. Plyometric training in isolation seems to be effective as well, although the ideal scenario is its incorporation within a multicomponent program.

### 4.2. Ankle Sprain

The results of the review show data on training methods and on the use of sports equipment.

In terms of training programs, two of the papers’ studies on analytical stability work of the ankle joint show positive results, with the use of semi-globes and exercises without material. Another of the programs, by Emery et al., in which the Wobble Board platforms are analyzed, did not obtain good results. However, in this last work, compliance with the program was low (298 out of 494 participants, or 60.3%), so this may be the cause of the ineffectiveness of the training [41]. Regarding neuromuscular training, of the two papers analyzed [39,47], only one was effective in reducing the incidence of sprains. Both programs included strength, running, plyometric, mobility, and agility exercises, but only the program by Stojanovic et al. included lower body stability exercises [47]. This could be the reason for the difference in the effectiveness of both programs. Therefore, solid evidence on neuromuscular training is lacking. Isolated ankle stability training appears to be the best preventive method.

On the other hand, regarding sports equipment, lace-up ankle braces may be a good option as seen in the results [44], although the type of shoe (low or high) seems to not influence the results [38]. Another method contemplated that could be effective is the use of a lateral patch (spraino low-friction shoe patch) that is placed on the shoe. This method has shown efficacy in a reduction in a sample composed of basketball, handball, and badminton players [52]. However, specific results for basketball players were not shown, so their evidence cannot be accepted in the present review since it does not meet the inclusion criteria.

It would be of great interest to analyze the joint efficacy of using analytical programs for ankle stability, together with the use of ankle braces during the game. Another important aspect to consider is whether the ankle braces should always be used. Different studies show that the use of this implementation can modify both the range of motion (ROM) of the joint and the muscle electromyographic (EMG) activity of the stabilizing musculature [53,54], similar to what happens with ankle tapes [55,56]. If the constant use of both ankle braces and bandages reduces both the ROM and the EMG activity, the use of these methods may be inadvisable, or we should consider at what times they should be used. Perhaps it could be interesting to study whether it is convenient to use it only in matches, moments after an injury, or simply try to individualize each case.

In summary, training programs based on ankle joint stability and balance exercises are effective. The frequency of work is between 1 and 3 days per week, with a duration of between 5 and 20 min [40,49]. There is insufficient evidence to justify that multicomponent neuromuscular programs reduce the risk of ankle sprains, given that of the two studies analyzed only one showed positive results. With respect to other preventive strategies such as material or protections [3], it appears that ankle braces may be of great efficacy, as opposed to the type of footwear since the use of high-top or low-top shoes does not seem important in terms of reducing the risk of ankle sprains.

### 4.3. Anterior Cruciate Ligament

It seems that multicomponent programs [39,45], understood as neuromuscular training, are more effective in reducing ACL injury rates, as had been hypothesized [3]. The risk of suffering an ACL injury is multifactorial [18], so the approach from a neuromuscular program with different training content could try to reduce as many risk factors as possible. Against this, the program based on landings and decelerative movement mechanics included by Pfeiffer et al. did not obtain positive results [46]. This analytical approach may not be the most appropriate when multifactorial risk should be approached from a broader perspective.

There is no conclusive evidence regarding frequency and duration, but the study by Bonato et al. suggests performing the program as a warm-up for every training session, with a duration of 30 min (depending on the training days of each team), and the work by Omi et al. suggests a frequency of 3 days per week with a duration of 20 min [39,45]. Therefore, neuromuscular training programs for the prevention of ACL injuries should have a duration of 20–30 min being performed 3 days or more per week.

The three studies analyzed are composed of female samples, so the evidence applies to this population group of athletes. As discussed, ACL injury is much more prevalent in women [11], which probably arouses greater research interest as it is the major cause found in studies with samples of female athletes.

### 4.4. Sex

It appears that women have higher injury rates, both for general, ankle, and knee injuries, but there is no clear evidence regarding whether they respond better or worse to injury prevention programs based on neuromuscular training. Related to this, work conducted with players of different sports showed that women could respond worse to neuromuscular training programs [51]. Women do not seem to have higher injury rates, except for ACL injuries, as shown in the meta-analysis by Zech et al. [57]. Therefore, the differences in injury rates between men and women may be due to a worse response to neuromuscular training programs.

With regard to ankle sprains, with the data from the present review, it appears that ankle stability training programs are effective for both men and women. However, this cannot be determined for the use of ankle braces, since the only paper that discusses this does not show the results separated by sex.

As raised in the introduction, women tend to have a higher risk of ACL injury, and as explained above, the included studies do not include men in the sample.

### 4.5. Age

It can be determined that neuromuscular training is effective for injury prevention in both young player populations and senior athletes given the data collected in the present review [37,39,43,47,48]. With regard to a program based on plyometrics and landing technique, no differences can be extracted, nor can different age groups be compared for the efficacy of this type of training [36].

The work of Eils et al. only uses senior athletes, and the work of Cumps et al. includes university athletes as well as others in training age, although the mean age of the intervention group is 17.7 years [40,49]. Therefore, training based on stabilization exercises seems to be effective in the prevention of ankle sprains, regardless of age, although the evidence is not strong.

Regarding ACL prevention programs, it is not possible to compare the efficacy of neuromuscular programs between training and senior athletes, since the studies analyzed do not include a sample of young athletes.

In summary, there is no scientific evidence to justify whether school-age athletes respond better or worse to prevention strategies compared to professional athletes.

### 4.6. Methods and Other Injury Prevention Strategies

The studies analyzed have not addressed other measures discussed such as rule modification or training load control [3]. The modification of regulations does not appear in any of the analyzed studies, neither those excluded by inclusion criteria nor those discarded. However, load control is mentioned in many of the discarded articles and in some of those not included due to non-compliance with the criteria. The main problem found is that the studies that analyze the relationship between load and injury incidence are descriptive. Although the relationships between training and/or competitive load and injuries are high and significant [20,58], there are no intervention studies that have shown reductions in incidence when load control is carried out versus control groups or other periods of time in which it is not controlled.

Therefore, the present review only supports training methods and the use of ankle braces, but not other types of prophylactic measures or strategies.

### 4.7. Neuromuscular Training Effects

It has been demonstrated that various training methods exert a protective effect on injuries. This may be attributed to the acute or chronic effects that exercise can induce.

Neuromuscular training may induce acute physiological effects. These acute effects may contribute to creating an enhanced state of sports performance that ensures the athlete’s success. The mobility exercises performed as part of neuromuscular warm-ups allow for an increase in the range of motion and elevation of muscle temperature [59]. Another effect achieved through warm-ups is the enhancement of muscular activity. This can be explained by various factors. Improved activity of type II muscle fibers is facilitated by better utilization of phosphocreatine (PCr). There is also enhanced muscle contraction conduction and increased efficiency of nerve impulses due to post-activation performance enhancement (PAPE). These mechanisms might be accountable for the reduction in injury risk. Strength, plyometric, and agility exercises, such as jumping and sprinting, influence the activation of these mechanisms [60]. Notably, to enhance PAPE, incorporating exercises with heavy weights or maximum isometric contractions could be beneficial [60]. However, this kind of load has not been used in the gathered studies. It has been proven that core exercises enhance stability through improved neuromuscular performance [61]. Another benefit or acute effect attributed to neuromuscular training is the improvement in stability in the single-leg squat test, along with a reduction in dynamic knee valgus, a key risk factor for ACL injury [62]. The improvement in both general and single-leg stability may be attributed to enhanced muscle coactivation, facilitated by improved electrical activity [63].

On the other hand, effects achieved through training can be chronic, meaning that the benefit of reducing the risk of injury is attained over the medium to long term. A review examining the effects of various training programs incorporating FIFA 11+ revealed several benefits, including improvements in concentric and eccentric strength, enhanced agility in various tests, improved core muscle function, reduced dynamic knee valgus, increased ankle and hamstring mobility, sprinting ability, and coordination [64]. Another review examining neuromuscular training programs showed improvements in stability, agility, explosive strength, proprioceptive capacity, sprinting, and jumping [65]. The enhancement of athletic performance across different physical capacities may contribute to a reduction in the risk of injury.

## 5. Conclusions

Neuromuscular training programs that include various training contents, such as mainly strength, stability and core work, mobility, and agility, reduce the risk of general injuries in basketball players of any age and sex. For the program to be effective it should have a minimum frequency of 3 days a week, with 20 min of duration, up to a maximum of 30 min performed in each training session, being effective in both young and adult athletes. These same programs are effective for the reduction of ACL injuries in adult women.

In the case of ankle sprains, specific programs focused on ankle joint balance, control, and stability are effective in adult and young players for the prevention of this type of injury, with a frequency of 1–3 days a week and a minimum duration of 5 min and a maximum of 20 min. The use of ankle braces with laces is also effective.

For training programs to be effective, it is essential to maintain them over time and to achieve adherence to them. One of the most appropriate strategies is to use the programs as a warm-up or as part of it.

## 6. Practical Applications

Basketball teams should include in their warm-up routine, both for training and games, an exercise program that includes strength, stability or core, mobility, and agility work, as well as specific analytical exercises for ankle stabilization (see Table 2). This program should aim to reduce the incidence of injury and should be complementary to training that aims to improve the technical, tactical, and physical performance of the players. The use of ankle braces with laces can be a recommended strategy to be used in matches.

## Figures and Tables

**Table 1:** Summary of included articles.

Author	Aim	Sample	Intervention Protocol	Outcome Measurement	Conclusion
Aerts et al. [36]	To evaluate the applicability of a jump-landing training program in basketball players using RE-AIM.	24 teams from the Belgian national second division and regional divisions in Flanders (Belgium) aged between 15 and 41, male (*n* = 129) or female (*n* = 114) athletes.	3-month exercise program based on jumping and landing 2 days a week for 10 min. 5 plyometric/strength body weight exercises x 8–10 reps each day.	Injury incidence in the control group was higher (rate = 5.4/1000 h) than in the intervention group (rate = 3.6/1000 h) (HR = 0.40 [95% CI = 0.16–0.99]).	It is an effective program for the prevention of lower limb injuries in basketball players.
Emery et al. [41]	To examine the effectiveness of a stability training program in reducing injuries in adolescent basketball players.	920 basketball players between 12 and 18 years of age, male (*n* = 464) and female (*n* = 456).	5 min stabilization exercise program during warm-ups using Wobble Board equipment each training day. 20 min home workouts, 5 days a week, for 1 year.	The protective effect found with regard to all injuries (RR = [0.8 95% CI; 0.57–1.11]), lower extremity injury (RR = 0.83 [95% CI; 0.57–1.19]), and ankle sprain injury (RR = 0.71 [95% CI; 0.45–1.13]) were not statistically significant. Compliance with the program was low (298/494 or 60.3%).	Basketball-specific balance training program was effective in reducing acute injuries in high school basketball.
Barber-Foss et al. [37]	To determine the effects of a neuromuscular training program in high school and middle school athletes in 3 sports (basketball, volleyball, and soccer), both in all injuries and in knee and ankle injuries.	474 girls (222 middle school, 252 high school; age = 14.0 ± 1.7 years, height = 161.0 ± 8.1 cm, weight = 55.4 ± 12.2 kg)	Neuromuscular training program 2–3 sessions per week of 10–25 min 13 bodyweight core, stabilization, and strength exercises 8–15 reps, 1–2 sets.	Regarding the basketball group: The overall injury incidence was lower for the intervention group (rate = 4.99/1000 h) than in the control group (rate = 7.22/1000 h). ARR = 40 (95% CI = 0.92, 4.54). Specific injury data for each sport are not displayed.	Neuromuscular core and stabilization training has a beneficial protective effect on injury incidence, demonstrated at all levels of competition.
Cumps et al. [40]	To examine the efficacy of a 22-week stability and balance training program on the incidence of lateral ankle sprains.	54 athletes, male (*n* = 37) and female (*n* = 17) (control, *n* = 27, 18.0 ± 2.7 years; intervention, *n* = 26, 17.7 ± 3.9 years).	22-week training program using semi-globes (stabilization semi-globes), 3 days a week. 4 exercises each day, with variations in different positions Dribbling, passing, standing, and Aberdeen exercises on a bosu.	No significant differences were found, 3.54/1000 in the control group (95% CI: 1.23–5.85) and 1.19/1000 (95% CI: 0.15–2.25) in the intervention group. Analyzing the relative risk, significant differences were found in the intervention group with respect to the control group (RR = 0.30 [95% CI: 0.11–0.84]) and also in men (RR = 0.29 [95% CI: 0.09–0.93]).	Balance or stability training should be included as a part of the basketball players’ routine 2–3 times a week with a duration of 5 to 15 min.
Eils et al. [49]	To investigate the effectiveness of a multi-station proprioceptive training program in reducing the incidence of ankle sprains.	172 athletes (103 men and 69 women) from the seventh to the first German basketball division (professionals).	Ankle stability training (balance, passing ball, landing, single-leg jumping) Once a week and consisted of 6 exercises, for 20 min.	Higher incidence in the control group (1.66/1000 h vs. 4.69/1000 h, *p* = 0.012). Logistic regression was used to evaluate the risk of injury in the intervention group with respect to the control group. Odds ratio (OR) = 0.355 for the intervention group (OR = 0.355, [95% CI = 0.151–0.835, *p* = 0.018]).	A multi-station proprioceptive training program reduces the incidence of ankle sprains.
Bonato et al. [39]	To evaluate the effectiveness of a neuromuscular training program in elite female basketball players.	160 female Italian players over 18 years of age from a total of 15 teams.	Neuromuscular training with body weight, 2–3 sets. 4 general strength exercises, 2–3 sets, 5–12 reps, or 30″ isometric. 4 plyometric exercises, 1–3 sets, 12–20 reps. Speed and agility exercises, 4 basketball courts.	Injury incidence was lower for the experimental group with respect to the control group (rate = 1.66 vs. 4.69). There were significant differences for knee sprains (*p* = 0.037) and ACL (*p* = 0.038). There were no significant differences for ankle sprains (*p* = 0.507) and other types of discomfort such as muscular pain, lumbar pain, etc.	Self-weight neuromuscular training included in warm-up routines reduces the incidence of injury in elite female basketball players.
McGuine et al. [44]	To evaluate whether lace-up ankle braces reduce the incidence and severity of ankle sprains in high school basketball players.	The sample was 1460, male (*n* = 724) and female (*n* = 736) high school basketball players.	Participants wore lace-up anklets throughout the 2009–2010 season.	The incidence was higher in the control group (rate = 1.41/1000 h) than in the experimental group (rate = 0.47/1000 h) (HR = 0.32 (95% CI = 0.20–0.52; *p* < 0.01]). For players with previous sprains, the prevalence was higher in the control group (rate = 1.79/1000 h) than in the experimental (rate = 0.83/1000 h) (HR = 0.39 [95% CI = 0.17–0.90; *p* < 0.01])	The use of lace-up ankle braces reduces the incidence, but not the severity, of ankle sprains in both previously uninjured basketball players and those who have already suffered an ankle sprain.
Barret et al. [38]	To determine the influence of shoe type on the incidence of ankle sprains.	569 college basketball players (91.7% men and 8.3% women)	Use of the assigned shoes throughout the season. High-top shoes or low-top shoes.	The incidence was 4.80/1000 h in high-top shoes, 4.06 × 10/1000 h in low-top shoes, and 2.69/1000 h in high-top shoes with inner tubes. There were no significant differences.	There is no strong relationship between the type of athletic footwear and the incidence of ankle sprains.
Longo et al. [43]	To evaluate the effectiveness of the FIFA 11+ program in the prevention of injuries in basketball players.	The sample consisted of 121 male players of all categories from U12 to senior.	FIFA 11+ program in warm-ups for 9 months. 5 running technique exercises 6 strength exercises, 2–3 sets × 10 reps, or 20–30 s 3 jumping exercises, 3 sets each one across the court. 3 single-leg balance exercises x 2–3 sets each one	In the intervention group, injury rates were lower than those in the control group for general (0.95 vs. 2.16; *p* = 0.0004), training (0.14 vs. 0.76; *p* = 0. 007), lower extremity (0.68 vs. 1.4; *p* = 0.022), acute (0.61 vs. 1.91; *p* < 0.0001), and severe (0 vs. 0.51; *p* = 0.004), for trunk (0. 07 vs. 0.51; *p* = 0.013), leg (0 vs. 0.38; *p* = 0.007), and hip and groin (0 vs. 0.25; *p* = 0.023) compared to the control group.	Although the rate of knee and ankle injuries was not reduced, FIFA 11+ was able to reduce the severity of such injuries and the prevalence of overall and general lower extremity injuries. The FIFA 11+ warm-up program is effective in preventing injuries in elite male basketball players.
Pfeiffer et al. [46]	To test the effect of a knee ligament injury prevention program with respect to the incidence of non-contact ACL injuries.	The sample consisted of 1439 female high school soccer, basketball, and volleyball players.	Performed a plyometric-based training program twice a week throughout the season. 4–6 plyometric exercises each session.	In the case of female basketball players, there was a very similar incidence between the control group (rate = 0.111/1000 h) and the experimental group (0.167/1000 h), and the difference was not significant. The odds ratio for all sports was not significant (OR = 2.05, *p* > 0.05), although the study does not present specific data for female basketball players.	A 20 min program based on plyometrics, landing, and decelerative mechanics performed twice a week does not reduce the risk of ACL injury in high school athletes.
Omi et al. [45]	To determine the effect of a training program based on joint work on ACL injury incidence in female basketball players.	The sample was 309 female basketball players, NCAA Division II college athletes.	Incorporated a 3-day-a-week body weight training program. 5 strength exercises, 2 sets × 10–20 reps, or 30″ isometric 4 jump landing exercises x 10 reps. 2 balance exercises x 2 sets × 20–30 reps.	The incidence of all ACL lesions was higher in the observation period (rate = 0.25/1000 h) than in the intervention period (rate = 0.1/1000 h). The relative risk was much lower (RR = 0.38; 95% CI, 0.17–0.87; *p* = 0.017) with ARR (0.032, 95% CI, 0.027–0.037) and NNT (31.6, 95% CI, 27.1–37.7). With respect to non-contact ACL injuries, the incidence in the intervention period was lower (rate = 0.08/1000 h) than in the observation period (rate = 0.21/1000 h) with a relative risk reduction (RR = 0.37; 95% CI, 0.15–0.92; *p* = 0.026), and with ARR and NNT values of 0.024 (95% CI, 0.020–0.029) and 41.3 (95% CI, 34.6–51.3), respectively.	A hip-focused training program demonstrates significant reductions in the incidence of injury in female basketball players.
Hewett et al. [42]	To evaluate the effect of a neuromuscular training program on the incidence of knee injuries in female soccer, basketball, and volleyball players.	The basketball sample consisted of 273 female players, all of whom were of high school age.	Program based on plyometrics during the preseason. 7–9 plyometric exercises, 20–30 s, or 5–10 reps	Examining non-contact injuries, a trend (*p* = 0.019) emerged, suggesting fewer injuries among trained female basketball players compared to those without training. Notably, the incidence of non-contact injuries in trained female athletes closely resembled that observed in males.	There are fewer non-contact injuries in female basketball players after performing a plyometric-based neuromuscular training program.
Stojanovic et al. [47]	To analyze the effect of a warm-up based on neuromuscular training on the prevention of lower limb injuries.	The sample consisted of 57 players (male: *n* = 42; female: *n* = 15) between 18 and 29 years old in the regional category.	The warm-up combined the following: 5 running technique exercises x 2 sets. 2 agility exercises x 2 sets. 10 plyometric, balance, and strength exercises, 2 sets each one.	The intervention group experienced a significantly lower incidence rate of ankle sprain (IRR = 0.26, 95% CI = 0.05, 0.98, *p* = 0.02) and a lower incidence rate of knee injury (IRR = 0.32, 95% CI = 0.03, 1.78, *p* = 0.07) compared to the control group. With respect to general non-contact injuries, the intervention group experienced a significantly lower incidence rate compared to the control group (IRR = 0.26, 95% CI = 0.05, 0.98, *p* < 0.001).	Warm-up based on a multicomponent training program reduces the risk of lower limb injuries, more specifically ankle sprains and knee sprains in basketball players.
Emery et al. [48]	To evaluate the effectiveness of a warm-up based on neuromuscular training on the incidence of ankle and knee injuries in basketball players.	Players between 11 and 18 years old from sixty-three teams (male: *n* = 442; female: *n* = 367).	The training program combined the following: 4 endurance exercises, 2 agility exercises, 5 strength exercises, and 2 balance exercises.	The SHRed injuries basketball program was effective against knee and ankle injuries (IRR = 0.64; 95% confidence interval (CI): 0.51, 0.79). There was no significant difference between the unsupervised group (IRR = 0.62; 95% CI: 0.47, 0.83) and the supervised group (IRR = 0.64; 95% CI: 0.49, 0.85).	The SHRed injuries basketball program is associated with a 36% reduction in injury incidence. Neuromuscular training programs for basketball players are recommended as a minimum standard to be met.

ACL = anterior cruciate ligament; ARR = Absolute Risk Ratio; CI = confidence interval; cm = centimeters; HR = hazard ratio; h = hour; IRR = Incidence Rate Ratio; NCAA = National Collegiate Athletic Association; NNT = Number Needed to Treat; OR = odds ratio; reps = repetitions; RR = risk ratio; s = seconds.

**Table 2:** Warm-up program.

**Part 1. Mobility (about 2–3 min)**
90–90 hip (1 set × 8 reps) Half kneeling ankle mobility (1 set × 8 reps each side) Half kneeling hamstring mobility (1 set × 8 reps each side) Squat with thoracic rotation (1 set × 6 reps each side) Cat–camel (1 set × 8 reps)
**Part 2. Strength, core, and ankle stabilization (about 5–7 min)**
6. Single-leg deadlift (2 sets × 8 reps each side) 7. Isometric lunge (2 sets × 15″ seconds each side) 8. Miniband monster walk (1 set × 6 steps each side) 9. Miniband front raises pulses (1 set × 4 reps) 10. Overhead Pallof press in pairs with teammate (1 set × 5 reps each side) 11. Single-leg stabilization passing with teammate (1 set × 8 reps each side) 12. Bear crawl shoulder touches (2 sets × 8 reps each side) 13. Abduction side plank (1 set × 10–15″ seconds each side) 14. Skater jumps (2 sets × 6 reps each side)
**Part 3. Agility (running technique drills and plyometrics) (about 4–6 min)**
15. Skipping (1 set, jog back) 16. Russian skipping (1 set, jog back) 17. Combined skipping: 1 leg skipping, 1 leg Russian skipping (1 set each leg, jog back) 18. Lateral “shuffle” step (1 set each leg, jog back) 19. Lateral “cross” step (1 set each leg, jog back) 20. Running backward (1 set, jog back) 21. Ankle jumps (2 sets, jog back) 22. Start from plyo step + sidestep (1 set each side, jog back) 23. Start from hip turn + sidestep (1 set each side, jog back) 24. Horizontal jumps (1 set × 6 reps.) 25. Single-leg hop jumps (1 set each leg × 5 jumps)

## Appendix A

**Table A1:** RE-AIM.

First Author, Reference	Aerts [36]	Emery [41]	Barber—Foss [37]	Cumps [40]	Eils [49]	Bonato [39]	McGuine [44]	Barrett [38]	Longo [43]	Pfeiffer [46]	Omi [45]	Hewett [42]	Stojanovic [47]	Emery [48]
Did the study report the % of potential participants who were excluded OR the characteristics of participants who were excluded?	Yes	Yes	No	No	Yes	Yes	No	Yes	Yes	No	No	No	No	Yes
Was the % of individuals participating based on a valid denominator reported [not volunteers interested]?	Yes	Yes	Yes	No	Yes	Yes	Yes	Yes	Yes	Yes	No	No	Yes	No
Were the characteristics of the participants compared to non-participants or to the target population?	Yes	No	Yes	No	Yes	Yes	Yes	Yes	No	No	Yes	No	Yes	Yes
Was a measure of the primary outcome with or without comparison to a public health goal reported?	Yes	Yes	Yes	Yes	Yes	Yes	Yes	Yes	Yes	Yes	Yes	Yes	Yes	Yes
Was any within-group analysis conducted that allowed researchers to draw conclusions about how different subgroups responded?	No	Yes	Yes	No	No	No	No	Yes	No	Yes	No	Yes	No	Yes
Did the study report the % of potential settings that were excluded OR reasons for exclusions?	Yes	Yes	No	No	No	No	Yes	No	No	No	No	No	No	No
Did the study report the % of settings accepting participation? The denominator should not be volunteers indicating interest.	Yes	Yes	No	No	No	No	Yes	No	Yes	No	No	No	No	No
Were the characteristics of those settings choosing to participate and those unwilling to participate described?	Yes	No	No	No	No	Yes	No	No	No	No	No	No	No	No
Was the % of perfect delivery or sessions completed reported [e.g., adherence or consistency]?	Yes	No	Yes	Yes	No	Yes	No	Yes	Yes	No	Yes	No	Yes	Yes
Was a measure of the primary outcome [with or without comparison to a public health goal] at >6 months after the final intervention by the study’s researchers reported?	No	No	No	No	No	No	No	No	No	No	No	Yes	No	No
**TOTAL**	**8**	**6**	**5**	**2**	**4**	**6**	**5**	**6**	**5**	**2**	**3**	**3**	**4**	**5**

## References

[1] Andreoli C.V., Chiaramonti B.C., Buriel E., Pochini A.D.C., Ejnisman B., Cohen M. (2008). Epidemiology of sports injuries in basketball: Integrative systematic review. BMJ Open Sport Exerc. Med..

[2] Herzog M.M., Mack C.D.F., Dreyer N.A., Wikstrom E.A., Padua D.A., Kocher M.S., Di Fiori J.P., Maarshall S.P. (2019). Ankle Sprains in the National Basketball Association, 2013–2014 through 2016–2017. Am. J. Sports Med..

[3] Emery C.A., Pasanen K. (2019). Current trends in sport injury prevention. Best Pract. Res. Clin. Rheumatol..

[4] Barden D.C., Thain D.P.K. (2022). Injury Surveillance in English Youth Basketball: A 5-season Cohort Study to Inform Injury Prevention Strategies. Phys. Ther. Sport.

[5] Vázquez-Guerrero J., Fernández-Valdés B., Jones B., Moras G., Reche X., Sampaio J. (2019). Changes in Physical Demands Between Game Quarters of U18 Elite Official Basketball Games. PLoS ONE.

[6] Gonzalo-Skok O. (2015). La Velocidad en el Cambio de Dirección en los Deportes de Equipo: Evaluación, Especificidad y Entrenamiento. Ph.D. Thesis.

[7] Verschueren J., Tassignon B., De Pauw K., Proost M., Teugels A., Van Cutsem J., Roelands B., Verhagen E., Meeusen R. (2020). Does Acute Fatigue Negatively Affect Intrinsic Risk Factors of the Lower Extremity Injury Risk Profile? A Systematic and Critical Review. Sports Med..

[8] Clifton D.R., Onate J.A., Hertel J., Pierpoint L.A., Currie D.W., Wasserman E.B., Knowles T.P., Dompier T.P., Marshall S.W., Comstock R.D. (2018). The First Decade of Web-Based Sports Injury Surveillance: Descriptive Epidemiology of Injuries in US High School Boys’ Basketball (2005–2006 through 2013–2014) and National Collegiate Athletic Association Men’s Masketball (2004–2005 through 2013–2014). J. Athl. Train..

[9] Clifton D.R., Hertel J., Onate J.A., Currie D.W., Pierpoint L.A., Wasserman E.B., Knowles T.P., Dompier T.P., Comstock R.D., Marshall S.W. (2018). The First Decade of Web-Based Sports Injury Surveillance: Descriptive Epidemiology of Injuries in US High School Girls’ Basketball (2005–2006 Through 2013-2014) and National Collegiate Athletic Association Women’s Basketball (2004–2005 Through 2013–2014). J. Athl. Train..

[10] Sánchez Jover F., Gómez Conesa A. (2008). Epidemiology of Sports Injuries Basketball. Rev. Int. Med. Cienc. Act. Fís. Deporte.

[11] Stojanović E., Faude O., Nikić M., Scanlan A.T., Radovanović D., Jakovljević V. (2023). The Incidence Rate of ACL Injuries and Ankle Sprains in Basketball Players: A Systematic Review and Meta-analysis. Scand. J. Med. Sci. Sports.

[12] Hewett T.E. (2000). Neuromuscular and Hormonal Factors Associated with Knee Injuries in Female Athletes: Strategies for Intervention. Sports Med..

[13] Kobayashi T., Tanaka M., Shida M. (2016). Intrinsic Risk Factors of Lateral Ankle Sprain: A Systematic Review and Meta-analysis. Sports Health.

[14] Grindem H., Snyder-Mackler L., Moksnes H., Engebretsen L., Risberg M.A. (2016). Simple Decision Rules Can Reduce Reinjury Risk by 84% After ACL Reconstruction: The Delaware-Oslo ACL Cohort Study. Br. J. Sports Med..

[15] Van Melick N., Van Cingel R.E.H., Brooijmans F., Neeter C., Van Tienen T., Hullegie W., Nijhuis-van der Sanden M.W.G. (2016). Evidence-Based Clinical Practice Update: Practice Guidelines for Anterior Cruciate Ligament Rehabilitation Based on a Systematic Review and Multidisciplinary Consensus. Br. J. Sports Med..

[16] Kobayashi H., Kanamura T., Koshida S., Miyashita K., Okado T., Shimizu T., Yokoe K. (2010). Mechanisms of the Anterior Cruciate Ligament Injury in sports Activities: A twenty-year Clinical Research of 1700 Athletes. J. Sports Sci. Med..

[17] Shimokochi Y., Shultz S.J. (2008). Mechanisms of Noncontact Anterior Cruciate Ligament Injury. J. Athl. Train..

[18] Smith H.C., Vacek P., Johnson R.J., Slauterbeck J.R., Hashemi J., Shultz S., Beynnon B.D. (2012). Risk Factors for Anterior Cruciate Ligament Injury: A Review of the Literature—Part 1: Neuromuscular and Anatomic Risk. Sports Health.

[19] Bahr R., Krosshaug T. (2005). Understanding Injury Mechanisms: A key Component of Preventing Injuries in Sport. Br. J. Sports Med..

[20] Lewis M. (2018). It’s a Hard-Knock Life: Game Load, Fatigue, and Injury Risk in the National Basketball Association. J. Athl. Train..

[21] Ferioli D., La Torre A., Tibiletti E., Dotto A., Rampinini E. (2021). Determining the Relationship Between Load Markers and Non-Contact Injuries During the Competitive Season Among Professional and Semi-Professional Basketball Players. Res. Sports Med..

[22] Gabbett T.J. (2016). The Training-Injury Prevention Paradox: Should Athletes be Training Smarter and Harder?. Br. J. Sports Med..

[23] Orringer M.J., Pandya N.K. (2022). Acutely Increased Workload is Correlated with Significant Injuries Among National Basketball Association Players. Int. J. Sports Sci. Coach..

[24] Bonazza N.A., Smuin D., Onks C.A., Silvis M.L., Dhawan A. (2017). Reliability, Validity, and Injury Predictive Value of the Functional Movement Screen. Am. J. Sports Med..

[25] Fuller J.T., Chalmers S., Debenedictis T.A., Townsley S., Lynagh M., Gleeson C., Zacharia A., Thomson S., Magarey M. (2017). High Prevalence of Dysfunctional, Asymmetrical, and Painful Movement in Elite Junior Australian Football players Assessed Using the Functional Movement Screen. J. Sci. Med. Sport.

[26] Moore E., Chalmers S., Milanese S., Fuller J.T. (2019). Factors Influencing the Relationship between the Functional Movement Screen and Injury Risk in Sporting Populations: A Systematic Review and Meta-analysis. Sports Med..

[27] Moran R.W., Schneiders A.G., Mason J., Sullivan S.J. (2017). Do Functional Movement Screen (FMS) Composite Scores Predict Subsequent Injury? A Systematic Review with Meta-Analysis. Br. J. Sports Med..

[28] Okada T., Huxel K.C., Nesser T.W. (2011). Relationship between Core Stability, Functional Movement, and Performance. J. Strength Cond. Res..

[29] Yin L., Mancheno-Unda M.A., Li S. (2023). Application of Multi-Target Positioning Algorithm in Basketball Teaching System and Injury Prevention. Prev. Med..

[30] Xu T., Tang L. (2021). Adoption of Machine Learning Algorithm-Based Intelligent Basketball Training Robot in Athlete Injury Prevention. Front. Neurorobot..

[31] Ang Z. (2023). Application of IoT Technology based on Neural Networks in Basketball Training Motion Capture and Injury Prevention. Prev. Med..

[32] Chen Z.H., Zhang G.Q. (2023). CNN Sensor Based Motion Capture System Application in Basketball Training and Injury Prevention. Prev. Med..

[33] Moher D., Liberati A., Tetzlaff J., Altman D.G., Altman D., Antes G., PRISMA Group (2009). Preferred Reporting Items for Systematic Reviews and Meta-Analyses: The PRISMA Statement. PLoS Med..

[34] Glasgow R.E., Vogt T.M., Boles S.M. (1999). Evaluating the Public Health Impact of Health Promotion Interventions: The RE-AIM Framework. Am. J. Public Health.

[35] Taylor J.B., Ford K.R., Nguyen A.D., Terry L.N., Hegedus E.J. (2015). Prevention of Lower Extremity Injuries in Basketball: A Systematic Review and Meta-Analysis. Sports Health.

[36] Aerts I., Cumps E., Verhagen E., Mathieu N., Van Schuerbeeck S., Meeusen R. (2013). A 3-Month Jump-Landing Training Program: A Feasibility Study Using the RE-AIM Framework. J. Athl. Train..

[37] Barber-Foss K.D., Thomas S., Khoury J.C., Myer G.D., Hewett T.E. (2018). A School-Based Neuromuscular Training Program and Sport-Related Injury Incidence: A Prospective Randomized Controlled Clinical Trial. J. Athl. Train..

[38] Barrett J.R., Tanji J.L., Drake C., Fuller D., Kawasaki R.I., Fenton R.M. (1993). High- Versus Low-Top Shoes for the Prevention of Ankle Sprains in Basketball Players. A prospective Randomized Study. Am. J. Sports Med..

[39] Bonato M., Benis R., La Torre A. (2018). Neuromuscular Training Reduces Lower Limb Injuries in Elite Female Basketball Players. A cluster randomized controlled trial. Scand. J. Med. Sci. Sports.

[40] Cumps E., Verhagen E., Meeusen R. (2007). Efficacy of A Sports Specific Balance Training Programme on The Incidence of Ankle Sprains in Basketball. J. Sports Sci. Med..

[41] Emery C.A., Rose M.S., McAllister J.R., Meeuwisse W.H. (2007). A Prevention Strategy to Reduce the Incidence of Injury in High School Basketball: A Cluster Randomized Controlled Trial. Clin. J. Sport Med..

[42] Hewett T.E., Lindenfeld T.N., Riccobene J.V., Noyes F.R. (1999). The Effect of Neuromuscular Training on the Incidence of Knee Injury in Female Athletes. A prospective Study. Am. J. Sports Med..

[43] Longo U.G., Loppini M., Berton A., Marinozzi A., Maffulli N., Denaro V. (2012). The FIFA 11+ Program is Effective in Preventing Injuries in Elite Male Basketball Players: A Cluster Randomized Controlled Trial. Am. J. Sports Med..

[44] McGuine T.A., Brooks A., Hetzel S. (2011). The Effect of Lace-Up Ankle Braces on Injury Rates in High School Basketball Players. Am. J. Sports Med..

[45] Omi Y., Sugimoto D., Kuriyama S., Kurihara T., Miyamoto K., Yun S., Kawashima T., Hirose N. (2018). Effect of Hip-Focused Injury Prevention Training for Anterior Cruciate Ligament Injury Reduction in Female Basketball Players: A 12-Year Prospective Intervention Study. Am. J. Sports Med..

[46] Pfeiffer R.P., Shea K.G., Roberts D., Grandstrand S., Bond L. (2006). Lack of Effect of a Knee Ligament Injury Prevention Program on the Incidence of Noncontact Anterior Cruciate Ligament Injury. J. Bone Jt. Surg. Am..

[47] Stojanović E., Terrence-Scanlan A., Radovanović D., Jakovljević V., Faude O. (2023). A Multicomponent Neuromuscular Warm-Up Program Reduces Lower-Extremity Injuries in Trained Basketball Players: A Cluster Randomized Controlled Trial. Phys. Sportsmed..

[48] Emery C.A., Owoeye O.B.A., Räisänen A.M., Befus K., Hubkarao T., Palacios-Derflingher L., Pasanen K. (2022). The “SHRed Injuries Basketball” Neuromuscular Training Warm-up Program Reduces Ankle and Knee Injury Rates by 36% in Youth Basketball. J. Orthop. Sports Phys. Ther..

[49] Eils E., Schröter R., Schröderr M., Gerss J., Rosenbaum D. (2010). Multistation Proprioceptive Exercise Program Prevents Ankle Injuries in Basketball. Med. Sci. Sports Exerc..

[50] Munoz-Plaza C., Pounds D., Davis A., Park S., Sallis R., Romero M.G., Sharp A.L. (2021). High School Basketball Coach and Player Perspectives on Warm-Up Routines and Lower Extremity Injuries. Sports Med. Open.

[51] Raäisaänen A.M., Galarneau J.M., Van Den Berg C., Eliason P., Benson L.C., Owoeye O.B.A., Pasanen K., Hagel B., Emery C.A. (2023). Who Does Not Respond to Injury Prevention Warm-up Programs? A Secondary Analysis of Trial Data from Neuromuscular Training Programs in Youth Basketball, Soccer, and Physical Education. J. Orthop. Sports Phys. Ther..

[52] Lysdal F.G., Bandholm T., Tolstrup J.S., Clausen M.B., Mann S., Petersen P.B., Grønlykke T.N., Kersting U.K., Delahunt E., Thorborg K. (2021). Does the Spraino Low-Friction Shoe Patch Prevent Lateral Ankle Sprain Injury in Indoor Sports? A Pilot Randomised Controlled Trial with 510 participants with Previous Ankle Injuries. Br. J. Sports Med..

[53] Barlow G., Donovan L., Hart J.M., Hertel J. (2015). Effect of Lace-Up Ankle Braces on Electromyography Measures During Walking in Adults with Chronic Ankle Instability. Phys. Ther. Sport.

[54] Luo Y., Hu M., Li Z., Huang X., Wu D., Li F., Wang S. (2023). Effect of Lace-Up Ankle Brace on the Tibiotalar and Subtalar Joint During the Landing. Front. Bioeng. Biotechnol..

[55] Biz C., Nicoletti P., Tomasin M., Bragazzi N.L., Di Rubbo G., Ruggieri P. (2022). Is Kinesio Taping Effective for Sport Performance and Ankle Function of Athletes with Chronic Ankle Instability (CAI)? A Systematic Review and Meta-Analysis. Medicina.

[56] Jeffriess M.D., Schultz A.B., McGann T.S., Callaghan S.J., Lockie R.G. (2015). Effects of Preventative Ankle Taping on Planned Change-of-Direction and Reactive Agility Performance and Ankle Muscle Activity in Basketballers. J. Sports Sci. Med..

[57] Zech A., Hollander K., Junge A., Steib S., Groll A., Heiner J., Nowak F., Pfeiffer D., Rahlf A.L. (2022). Sex Differences in Injury Rates in Team-Sport Athletes: A Systematic Review and Meta-Regression Analysis. J. Sport Health Sci..

[58] Weiss K.J., Allen S.V., McGuigan M.R., Whatman C.S. (2017). The Relationship Between Training Load and Injury in Men’s Professional Basketball. Int. J. Sports Physiol. Perform..

[59] Opplert J., Babault N. (2018). Acute Effects of Dynamic Stretching on Muscle Flexibility and Performance: An Analysis of the Current Literature. Sports Med..

[60] McGowan C., Pyne D.P., Thompson K.G., Rattray B. (2015). Warm-Up Strategies for Sport and Exercise: Mechanisms and Applications. Sports Med..

[61] Saki F., Shafiee H., Tahayori B., Ramezani F. (2023). The Effects of Core Stabilization Exercises on the Neuromuscular Function of Athletes with ACL Reconstruction. Sci. Rep..

[62] García-Luna M.A., Cortell-Tormo J.M., García-Jaén M., Ortega-Navarro M., Tortosa-Martínez J. (2020). Acute Effects of ACL Injury-Prevention Warm-Up and Soccer-Specific Fatigue Protocol on Dynamic Knee Valgus in Youth Male Soccer Players. Int. J. Environ. Res. Public Health.

[63] Latash M.L. (2018). Muscle coactivation: Definitions, mechanisms, and functions. J. Neurophysiol..

[64] Asgari M., Nazari B., Bizzini M., Jaitner T. (2023). Effects of the FIFA 11+ Program on Performance, Biomechanical Measures, and Physiological Responses: A Systematic Review. J. Sport Health Sci..

[65] Akbar S., Soh K.G., Nasiruddin N.J.M., Bashir M., Cao S., Soh K.L. (2022). Effects of Neuromuscular Training on Athletes Physical Fitness in Sports: A Systematic Review. Front. Physiol..

